# Association of Iris Structural Measurements with Corneal Biomechanics in Myopic Eyes

**DOI:** 10.1155/2021/2080962

**Published:** 2021-12-31

**Authors:** Lin Fu, Yufeng Ye, Xu Jia, Yunjie Zhang, Xiaoyu Chen, Hengli Lian, Weihua Yang, Qi Dai

**Affiliations:** ^1^School of Ophthalmology and Optometry, Eye Hospital, Wenzhou Medical University, Wenzhou 325027, China; ^2^Wenzhou Medical University, Wenzhou 325027, China; ^3^Affiliated Eye Hospital, Nanjing Medical University, Nanjing 210029, China

## Abstract

**Purpose:**

To evaluate the relationship between iris sectional parameters on swept-source optical coherence tomography (SS-OCT) with corneal biomechanics measured by Corneal Visualization Scheimpflug Technology (Corvis ST) in young adults with myopia.

**Methods:**

117 patients with myopia aged ≥18 years were recruited from the Eye Hospital of Wenzhou Medical University, who had complete SS-OCT and Corvis ST data. Only the left eye of each participant was selected for analysis. Iris sectional parameters included iris thickness at 750 *μ*m from the scleral spur (IT750), iris sectional area (I-area), and iris curvature (I-curv) measured from four quadrants. Associations between the iris parameters and corneal biomechanics were analyzed using linear regression models.

**Results:**

The mean age of the included young adults was 26.26 ± 6.62 years old with 44 males and 73 females. The iris parameters were different among the four quadrants. The nasal, temporal, and inferior quadrants of IT750, together with nasal and temporal quadrants of I-area, were correlated with corneal biomechanical parameters after being adjusted for age, gender, pupil diameter, and axial length. Thicker IT750 and larger I-area were related to a softer cornea. However, no association was found between I-curv and corneal biomechanics.

**Conclusions:**

Iris sectional parameters measured from SS-OCT images were associated with corneal biomechanical properties in myopic eyes. Thicker IT750 and larger I-area indicate a softer cornea. IT750 and I-area may provide useful information on corneal biomechanical properties in myopic eyes.

## 1. Introduction

Myopia is one of the most common causes of preventable visual impairment and blindness, with a predicted prevalence of 50% in the world's population in 2050.[1]. High or pathological myopia can result in irreversible ocular blinding diseases, such as choroidal neovascularization, retinal detachment, and glaucoma [2–4]. This will definitely lead to heavy cost burden on the families and society. Together with the increased prevalence of myopia, there has been an urge to view this major public health problem and better understand the pathophysiology of myopia.

The development of myopia is believed to be induced by various patterns of ocular expansions, including equatorial expansion [5], axial expansion [6], and posterior pole expansion [7]. Accompanied by these expansions, the pathogenesis of myopia includes the biomechanical alternations of the eyeball outer wall, including the cornea, scleral, lamina cribrosa, and peripapillary ring [8, 9]. The corneal stiffness was reported to be reduced with the increase in the myopic degree from Corneal Visualization Scheimpflug Technology (Corvis ST, Oculus, Wetzlar, Germany) studies [10–12]. Accompanied by these expansions, the inner tissue especially the iris would also be changed. In a population of 18 to 66 years old, eyes with concave iris configuration are mostly from myopic eyes and no hyperopic eyes displayed a concave iris [13]. This suggests that the iris structural features may be related to myopia. However, the underlying mechanism so far is not clear. It may be related to the development and biomechanics of the cornea and sclera, the structure of the iris and choroid, and the microcirculation of blood vessels.

As iris stroma and corneal stroma are both of mesodermal origin [14, 15], in our previous work, we found that the iris surface features including crypts and furrows were associated with corneal biomechanical parameters [16]. We postulate that the iris structural features may also be related to corneal characteristics. With the advantages of SS-OCT and Corvis ST, we are aiming to explore the relationships of iris structural measurements with corneal biomechanics in myopic eyes in this study. This will enrich the understanding of the pathophysiology of myopia and may help to identify the risk factors of myopia.

## 2. Methods

### 2.1. Participants

In this prospective, cross-sectional study, myopic subjects were recruited from a population of refractive surgery candidates in Eye Hospital of Wenzhou Medical University which aged older than 18 years and without any ocular pathology other than refractive error. Data from the left eyes of all subjects were selected for analysis to eliminate an intereye correlation issue.

This study was conducted following the tenets of the Declaration of Helsinki and was approved by the Institutional Review Board of Wenzhou Medical University (IRB approval number 2020-128-K-113). This study was registered on Chinese Clinical Trial Registry with the registration number of ChiCTR2100052498. All the subjects signed an informed consent form at the time of recruitment.

### 2.2. Ocular Examinations

Detailed ophthalmological examinations were performed by an experienced ophthalmologist for all subjects, including slit-lamp examination, refraction measurement (spherical equivalence (SE)), best-corrected visual acuity, anterior segment images scanned by swept-source optical coherence tomography (SS-OCT, Casia SS-1000 OCT, Tomey, Nagoya, Japan), corneal tomography with the Pentacam (Oculus, Wetzlar, Germany), corneal biomechanical properties assessed by the Corvis ST (Oculus, Wetzlar, Germany), and ocular axial length measured by the IOL-Master (Carl Zeiss Meditec, Jena, Germany).

### 2.3. Iris Measurements from SS-OCT

**Figure 1:**
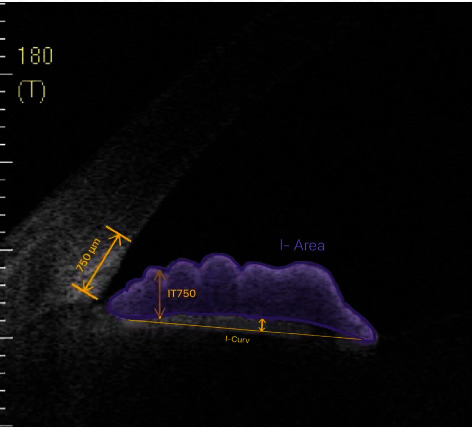
Measurement of the cross-sectional iris thickness, iris area, and iris curvature on anterior segment optical coherence tomography. IT750: iris thickness at 750 *μ*m from the scleral spur; I-area: iris area; I-curv: iris curvature.

All subjects underwent a standard swept-source optical coherence tomography (SS-OCT, Casia SS-1000 OCT, Tomey, Nagoya, Japan) examination by a single examiner who was masked to the clinical data. The horizontal and vertical scans of the nasal, temporal, superior, and inferior four quadrants of all the participants were obtained using SS-OCT under standardized dark condition (20 lux). Images with the best quality were obtained for the iris measurements. The iris thickness at 750 *μ*m from the scleral spur (IT750) and iris area (I-area) was measured as the cross-sectional area from the pupil to the scleral spur, and the iris curvature (I-curv) was measured as the distance from the greatest convexity to the line drawing from the most central to the most peripheral point of the iris pigment epithelium (Figure 1) as previously defined using ImageJ software [17, 18]. The mean values of the 4 quadrants were used for the analysis.

### 2.4. Corvis ST Measurement

The corneal biomechanical properties were obtained using the Corvis ST. Due to an airflow released by the instrument, the cornea generates an inward movement through the first applanation to the highest degree of concavity and then moves outward through the second applanation to the natural shape. In this process, 140 sequential horizontal images of the cornea were captured with a high-speed Scheimpflug camera at a speed of 4330 frames per second in 30 ms. Meanwhile, the biomechanical parameters were calculated by the device including bIOP; maximum DA (DA max) at the first applanation; time at the highest concavity (HC time); time at the first and second applanation (A1 time and A2 time, respectively); corneal velocity at the first and second applanation (A1 velocity and A2 velocity, respectively); DA; deflection length (DLL); deflection amplitude (DLA) and delta arc length (dArcL) at A1, HC, and A2; PD, radius, and maximum deflection amplitude at the first applanation (DLA Max); maximum delta arc length (dArcLM); maximum inverse radius (max inverse radius); maximum deformation amplitude ratio (DA ratio max) at 2 mm and 1 mm; central corneal thickness (CCT); integrated radius; and SP-A1.

### 2.5. Statistical Analysis

All statistical analyses were performed using SPSS version 23.0 software (SPSS for Windows, Chicago, IL, USA). Demographic data and ocular characteristics from the left eyes of the participants were described using the mean ± SD. One-way analysis of variance was used to compare the difference of iris measurements in 4 quadrants of left eyes. Linear regression models were performed to assess the associations between iris measurements (independent variable) and corneal biomechanical parameters measured by Corvis ST (dependent variable). They were adjusted for potential confounders such as age and gender in model 1 and additional ones of pupil diameters and axial length in model 2. A *P* < 0.05 was set to be statistically significant.

## 3. Results

In total, 18 subjects were excluded due to the following reasons: poor image quality (10), IOP or bIOP higher than 21 mmHg (5), and coexisting with posterior staphyloma (3). 117 patients were recruited for this study. The demographics and the characteristics of their ocular parameters are displayed in Table 1. Most of the included participants were young adults, and all of them were myopes. The mean values of corneal biomechanical parameters measured by the Dynamic Scheimpflug Analyzer (Corvis ST) are shown in Table 2.

**Figure 2:**
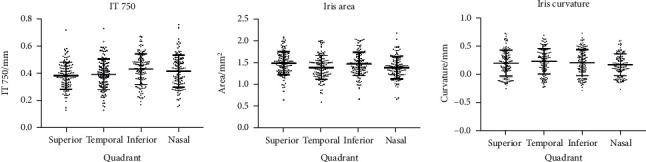
The distribution of iris parameters in four quadrants.

We measured the iris parameters in the superior, temporal, inferior, and nasal four quadrants (Figure 2). The IT750 was thicker in the inferior and nasal quadrants than in the superior and temporal quadrants (all *P* < 0.05). The IT750 in the inferior quadrant did not differ from the one in the nasal quadrant, and there were no differences of IT750 between the superior and temporal quadrants. The iris area in the superior and inferior quadrants was larger than that in the temporal and nasal quadrants (all *P* < 0.0001). For the iris configuration, the temporal iris was the most concave and the nasal iris was the least concave among the four quadrants (all *P* < 0.01). It was similar between the superior and inferior quadrants (*P* = 0.77). Moreover, the iris curvature was consistent among the four quadrants in most of the eyes except in one eye, and the iris was concave in the temporal quadrant and was convex in the other three quadrants. Therefore, in this population, there were 91 (77.8%) eyes in the superior, inferior, and nasal quadrants and 92 (78.6%) eyes in the temporal quadrant with the concave iris.

All quadrants of the IT750, iris area, and iris curvature were analyzed for the relationship with corneal biomechanical parameters. The correlated parameters are shown in Table 3. Our results showed that the correlation differs in different quadrants of the iris features.

For the IT750, temporal IT750 was related to more corneal biomechanical parameters than other quadrants. It was positively associated with PD, radius, HC DLL, and A2 DLL and negatively related to max inverse radius after being adjusted for age, gender, pupil diameter, and axial length in model 2. In addition, nasal IT750 was related to HC DLL, and inferior IT750 was associated with dArcLM positively in model 2. No correlation was found between superior IT750 and the corneal biomechanics.

For the iris area, there were two quadrants showing association with corneal biomechanical parameters in model 2. The nasal iris area was negatively related to the max inverse radius, and the temporal iris area was positively correlated with A2 time adjusted for age, gender, pupil diameter, and axial length. Although A2 velocity was negatively associated with the nasal iris area and inferior iris area in model 1 adjusted for age and gender, it showed no association with them after being adjusted for age, gender, pupil diameter, and axial length in model 2.

Finally, in model 2, the iris curvature demonstrated no correlation with all the corneal biomechanical parameters measured by Corvis ST. Only the inferior iris curvature showed a positive correlation with HC DLL when adjusting age and gender in model 1.

## 4. Discussion

In this cross-sectional study, we have provided new data on the distribution of iris structural parameters measured by SS-OCT including IT750, I-area, and I-curv in myopic patients and their associations with corneal biomechanics measured by Corvis ST. We showed that the iris parameters on SS-OCT differ among the ocular four quadrants in this myopic population. Although not all the quadrants of the iris parameters were related to corneal biomechanics, our results indicate that thicker IT750 and larger I-area in the nasal, temporal, and inferior quadrants were related to a softer cornea after being adjusted for age, gender, pupil diameter, and axial length.

IT750 and I-area are frequently studied in angle closure glaucoma and rarely described in myopes. Surprisingly, our results showed that IT750 and I-area were related to corneal biomechanical properties in this myopic population. I-area and IT750 are also the parameters associated with narrow angles diagnosed on gonioscopy [19]. Larger I-area and thicker IT750 are the risk factors for the narrow angle [19]. In the present study, these were related to a softer cornea. The determinant of angle width was always focused on the iris position, while the role of cornea in the angle width has not been studied. As part of the anterior chamber angle, corneal properties may also play an important role in the angle width. As greater I-area and IT750 were correlated with the softer cornea in this study, the softer cornea may also contribute to the narrow angle. The relationship between the iris and cornea demonstrated in our study provided further insights into the angle closure pathogenesis. Moreover, for the refractive surgery candidates, eyes with larger I-area and thicker IT750 may give the hint of a softer cornea to the surgeon.

Previously, iris structural parameters were widely studied in angle closure glaucoma, and the iris curvature was strongly related to angle width [20]. However, the role of the cornea in the angle width was neglected although the cornea is the outer border of angle width. Whether the corneal biomechanics influence the angle width and the iris contour is unknown, it is reported that corneal hysteresis (CH), a corneal biomechanical parameter measured from an ocular response analyzer (ORA), is associated with the scleral spur to spur distance [21, 22]. The cornea, scleral spur, and iris are all essential structures comprising the anterior chamber angle. As the neighboring structures, their relationships may play important roles in different ocular disorders.

The distribution of iris contour varies along age, gender, refraction, and different anterior segment diseases. Schuster et al. reported that none of the hyperopic eyes presented with a concave iris and eyes with a concave iris consisted of emmetropia and myopia [13]. In the report of narrow angles, all the iris was convex [19], and in eyes with pigment dispersion syndrome (PDS) or pigmentary glaucoma (PG), a concave iris is frequently observed [23]. The iris curvature reported in other literatures was mostly from temporal and nasal measurements in one meridian [19]. In the present study, we measured 4 quadrants of iris configuration from the vertical and horizontal meridian. There were 91 (77.8%) eyes in the superior, inferior, and nasal quadrants having a concave iris and 92 (78.6%) eyes in the temporal quadrant showing a concave iris. Our result was a reversal to the percentage of 26% concave iris and 67% convex iris in myopia from 560 eyes of 326 individuals in Schuster et al.'s report [13]. The reason may be that their age range was from 18 to 66 years and most of them were male. The age range in our study was 18 years to 40 years, and male consisted of 37.6% [13].

The major limitation of this study is that the study population only consisted of myopic patients and most of them were young age. Since the iris properties changed with age and refraction, the results in this study may not be able to be applied to other different populations. Further research may be necessary to conduct on people with emmetropia and hyperopia with a wider age range.

## 5. Conclusions

In conclusion, iris parameters measured by SS-OCT were associated with corneal biomechanics measured using Corvis ST. A larger iris sectional area and thicker IT750 were associated with a softer cornea in myopic eyes. The association between iris configuration and corneal biomechanics may help to understand the pathogenesis in several ocular disorders.

## Figures and Tables

**Table 1:** Demographics and baseline characteristics of participants (*n* = 117).

Characteristics	Mean (SD) or no. (%)
Age (yrs)	26.26 (6.62)
Gender	
Male	44 (37.6)
Female	73 (62.4)
Pupil diameter (mm)	3.05 (0.54)
ACD (mm)	3.70 (0.25)
Axial length (mm)	25.92 (1.13)
bIOP (mmHg)	15.41 (2.02)
CCT (*μ*m)	544.81 (34.18)
Refraction (SE)	-5.53 (2.22)
IT7500 (mm)	0.40 (0.09)
Iris area (mm^2^)	1.41 (0.24)
Iris curvature (mm)	0.19 (0.21)

ACD = angle chamber distance; CCT = central corneal thickness; SE = spherical equivalent; SD = standard deviation.

**Table 2:** Corneal biomechanical parameters measured by the Dynamic Scheimpflug Analyzer (*n* = 117).

Parameters	Mean (SD)
DA max (mm)	1.05 (0.09)
A1 time (ms)	7.44 (0.24)
A1 velocity (ms)	0.15 (0.02)
A2 time (ms)	22.00 (0.36)
A2 velocity (ms)	-0.27 (0.02)
HC time (ms)	16.66 (0.44)
PD (mm)	4.98 (0.23)
Radius (mm)	6.72 (0.66)
A1 DA (mm)	0.15 (0.01)
HC DA (mm)	1.05 (0.09)
A2 DA (mm)	0.32 (0.06)
A1 DLL (mm)	2.22 (0.23)
HC DLL (mm)	6.32 (0.78)
A2 DLL (mm)	2.89 (0.63)
A1 DLA (mm)	0.09 (0.01)
HC DLA (mm)	0.92 (0.09)
A2 DLA (mm)	0.10 (0.01)
DLA max (mm)	0.93 (0.09)
A1 dArcL (mm)	-0.02 (0.00)
HC dArcL (mm)	-0.13 (0.02)
A2 dArcL (mm)	-0.02 (0.01)
dArcLM (mm)	-0.15 (0.03)
Max inverse radius (mm^−1^)	0.18 (0.02)
DA ratio max (2 mm)	4.28 (0.34)
DA ratio max (1 mm)	1.56 (0.04)
Integrated radius (mm^−1^)	9.00 (0.90)
SP-A1	106.23 (17.83)

DA: deformation amplitude; A1: the first applanation; A2: the second applanation; HC: highest concavity; PD: peak distance; DLL: deflection length; DLA: deflection amplitude; dArcL: delta arc length; dArcLM: delta arc length max; SP-A1: stiffness parameter at the first applanation.

**Table 3:** Associations between iris surface parameters and corneal biomechanical parameters.

	Model 1^∗^		Model 2^ɫ^	
	*β*	*P* value	*β*	*P* value
Nasal IT750				
HC DLL	0.032	0.022	0.034	0.017
Nasal IT750				
PD	0.061	0.156	0.107	0.021
Radius	0.032	0.031	0.033	0.029
HC DLL	0.028	0.034	0.029	0.026
A2 DLL	0.036	0.021	0.034	0.027
Max inverse radius	-1.744	0.005	-1.825	0.004
Inferior IT750				
dArcLM	0.797	0.047	0.825	0.042
Nasal iris area				
A2 velocity	-2.430	0.016	-1.861	0.063
Max inverse radius	-2.696	0.074	-3.171	0.027
Temporal iris area				
A2 time	0.153	0.031	0.137	0.046
Inferior iris area				
A2 velocity	-2.386	0.024	-1.528	0.136
Inferior iris curvature				
HC DLL	0.052	0.045	0.048	0.064

^∗^Model 1 was adjusted for age and gender. ^†^Model 2 was adjusted for age, gender, pupil diameter, and axial length. HC: highest concavity; DLL: deflection length; PD: peak distance; A2: the second applanation; dArcLM: delta arc length max.

## Data Availability

The datasets used in this study are available based on reasonable request from the corresponding author Dr. Qi Dai, dq@mail.eye.ac.cn.
